# Approved Criminality

**Published:** 1892-07

**Authors:** J. M. Consley


### APPROVED CRIMINALITY.

BY PROF. J. M. CONSLEY.

By approved criminality we do not refer in the slightest degree to that class of sins which is looked on with favor by the immorally inclined portion of our race, but we wish to call attention to the criminality of the higher classes of our Christian people,—people who stand well before the world, in society and in church,—men and women who are called the bone and sinew of our social, moral and educational structures.

Almost every day we hear of some prominent person suddenly being called into eternity.

Apoplexy, heart disease or some such thing is assigned by our learned medical fraternity as the cause. Every day the number of persons of brilliant attainments who hurl themselves into eternity by the rash act of their own hand, is most marvelously increasing. "Temporary mental aberration" is assigned as the cause and we go on much as before. Who stops to inquire the cause of this apoplexy, heart disease, mental derangement, etc.? If they are becoming more common there must be a cause or combination of causes to produce such fatal results. And when a cause is found, that is capable of so cursing human-kind, will it not be a criminal thing?

It is a well-known fact that the diseases we have mentioned and many others of the same class are all traceable to some disordered state of the nervous system. Now nothing affects the nervous system so forcibly as *uncertainty* of mind. Next to this rank the cares and troubles incident to life. But the first named throws all others in the background in its baleful effects on the brain and nervous system generally. Right here comes the point to which we wish to call attention. Where is the limit of mental and physical endurance? (Not the *death*

limit, but the limit to which our powers may be taxed, without sinning against our bodily organism.) And is mankind, as a rule, keeping within the limit? I take my position on the negative side of the question and refer to the increase of sudden deaths to prove the correctness of the position taken.

The average wealth of the world has often been computed. The sum is not at all fabulous. In fact, it is considered paltry by the people of this nineteenth century. No man is considered wealthy until it takes six figures to represent his money. Now, if he can obtain this amount of money without injuring his health or any one else's comfort, all well and good. But if he can not, is he not committing a sin to attempt it? Any reasonable man will agree that he is. But there is such a general pell mell rush for wealth now, and in so many unscrupulous ways, that honest toil is too often deprived of its just earnings. This has become so common that the uncertainty of mind arising therefrom is having its name written in suicidal letters all over the world.

"Wisdom is the principal thing, therefore get wisdom," said the wisest of all men. But even the getting of wisdom has its limit; we endeavor to mark its limit in college and university courses and are rewarded by utter failure: for we find the standards too low for some minds, and so high that others can never reach them; while one now and then reaches the goal with an utterly worn out and worthless body that will last a year or two and then suddenly refuse to carry the disproportionate brain any longer. He won his honors with his life. Was it right? Have his instructors done their duty by him? His professor has no doubt held him up as an example to the dull and idle of the class thereby encouraging the self sacrifice. Could he not with the same observance that showed to him his pupil's wonderful mental development, see that his physical organism was being slowly burned out? Is a failure to observe these things excusable?

Then again in the strife for professional and political honors the limit of our powers is too often passed. In this field it seems to me there is more uncertainty than in any other. For here every passing breeze of public opinion has its effect. One moment a man may think he has almost attained the goal of his ambition, when like Tantalus' cup it recedes as he approaches and he sees, perhaps, a less worthy man fill the coveted position. The public is slow to see a man's talents and virtues until "every body" "says so," and then when that takes place he finds himself so suddenly thrust along, that success itself is almost as uncertain as the striving for it had been. And so he finds his life ground out between the will of the people and political machines and the duties of his office. But if all men would keep within their proper bounds in this strife would it be overly exhausting? I think not. If all would follow the teachings of the lowly Gallilean and not seek the "upper chambers" until bidden, political machines would starve to death and the "Will of the People" be transformed into the "Wish of the People." Then would this outcry in regard to the sudden deaths of prominent officials be abated, and they would have time to attend to their public duties and to take rest of both body and mind.

But in this age of steam and electricity, and labor saving devices for our hands and feet, our poor brains, the mainspring of our whole organisms, are alternately subjected to the white heat of this mad nineteenth century race, and the next deluged with the cold waves of uncertainty until they snap asunder. Life is gone and no one is blamed for it. But some one should be held responsible for his or her part in encouraging this self destruction. Will you and I stand altogether innocent of blame in the final reckoning?

